# Interplay between Path and Speed in Decision Making by High-Dimensional Stochastic Gene Regulatory Networks

**DOI:** 10.1371/journal.pone.0040085

**Published:** 2012-07-16

**Authors:** Nuno R. Nené, Alexey Zaikin

**Affiliations:** 1 Department of Mathematics, Imperial College London, London, United Kingdom; 2 Institute for Women’s Health and Department of Mathematics, University College London, London, United Kingdom; Humboldt University, Germany

## Abstract

Induction of a specific transcriptional program by external signaling inputs is a crucial aspect of intracellular network functioning. The theoretical concept of coexisting attractors representing particular genetic programs is reasonably adapted to experimental observations of “genome-wide” expression profiles or phenotypes. Attractors can be associated either with developmental outcomes such as differentiation into specific types of cells, or maintenance of cell functioning such as proliferation or apoptosis. Here we review a mechanism known as speed-dependent cellular decision making (SdCDM) in a small epigenetic switch and generalize the concept to high-dimensional space. We demonstrate that high-dimensional network clustering capacity is dependent on the level of intrinsic noise and the speed at which external signals operate on the transcriptional landscape.

## Introduction

**Figure 1:**
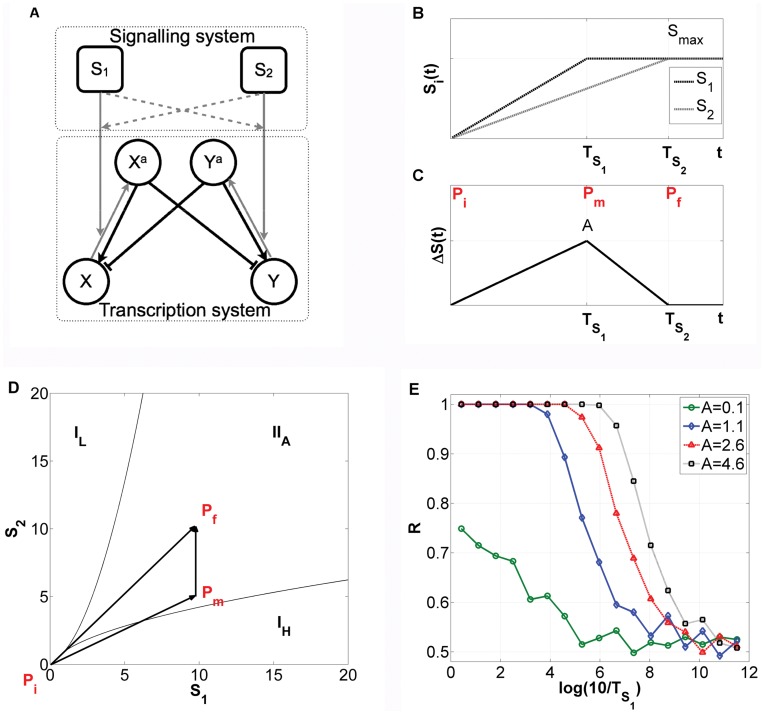
Paradigmatic integrated low order signaling–transcriptional circuit switch and speed-dependent cellular decision making. (A) Schematic representation: Nodes represent proteins, regulated by protein kinases with concentrations 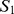 and 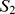, where 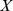 and 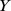 stand for transcription factors that can be phosporylated to generate 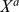 and 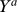. Black lines represent transcriptional interactions, while grey lines stand for protein-protein interactions. (B) Time evolution of the input signals 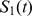 (black) and 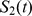 (grey), with 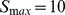. In [5] 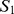 was considered to have a rising time 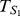 smaller than 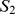. (C) Amplitude of the transient asymmetry between signals 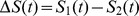. Here the maximal asymmetry is given by Eq. (1). (D) Phase diagram for 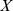 in the space (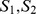). Thin lines represent borders between different regimes: 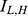 stands for monostability, with 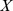 having a low or a high value, respectively. 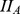 denotes bistability between two states at which 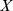 and 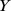 have opposite concentrations, (high, low) or (low, high). 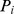, 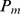 and 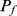 correspond to the initial (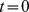), intermediary (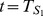), and final (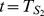) points of the signaling (see Fig. 1B and C). (E) Dependence of the fraction 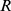 of cells that end up in the 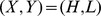, on the speed of the transition (measured by 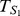) for different values of the maximum asymmetry A (see Fig. 1C). Noise intensity equals 0.01 for Fig. 1E, 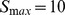 and there is no time scale difference between phosphorylation and transcription reactions. For further details see [5].



The conceptual framework of attractors in phase space representing particular transcriptional programs has been demonstrated in experimental observations of “genome-wide” expression profiles, e.g. in neutrophil differentiation [1], [2]. An attractor or dynamical regime is a stable solution to the set of mathematical equations that describe a dynamical system: that is, it represents the state of equilibrium to which a system will tend to move. Dynamical systems often have more than one solution, or attractor. In gene regulatory systems these can be either developmental outcomes such as specific types of differentiated cells, or maintenance of cell functioning such as proliferation or apoptosis. Each attractor, in normal circumstances, represents the adequate response to the combination of external signals and corresponds to a particular mRNA and protein concentration pattern [1]–[3]. Cell fate commitment has been correlated with both external signal duration and amplitude [4]. Additionally, the speed at which external signals induce changes on transcriptional landscapes has also recently been explored as an important mechanism for cell fate decision [5]. In fact, one of the mechanisms reported here explores this in connection with Speed-dependent Cellular Decision Making (SdCDM) observed in low order circuit models [5], but in a high-dimensional circuit. In Fig. 1 the main aspects of this mechanism are reviewed for the low order circuit explored in [5]. The combination of external signals 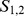 (see Fig. 1A and B) in the low order circuit takes the system from a state where the cell has only one possible end state (point 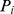), to a situation of bistability (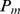), and finally to a point (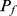) (see Fig. 1D) where the system ends up in one of two possible states. This constitutes the result of cellular decision making. Depending on the maximum of the time-dependent asymmetry between external signals (see Fig. 1C), the system will enter the bistability region at a different point of the 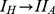 border (see Fig. 1D). Because the external signals end in the same values, one only has a transient asymmetry which biases the cellular decision making towards one of the available states in region 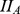. Therefore, the interval the system is exposed to that asymmetry influences the outcome of the decision. In the case of the simulations represented in Fig. 1, because 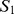 had always a smaller rising time (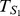) than 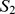 (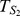), the final state selected with the highest probability was 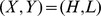 (H corresponds to high concentration values and L to low concentration values). The values of all parameters associated with transcription or translation processes were assumed to be symmetric in the circuit of Fig. 1A, in order to focus on the bias provided by external signals [5]. If the two signals 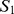 and 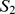 were identical and evolved in time at equal rates, the cell would undergo a transition to bistability through the straight line segment 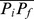. Along this segment there is complete symmetry, and consequently the cell would choose its fate stochastically between the two equally possible steady states. An interesting mechanism that was found in [5], the SdCDM effect (Fig. 1D), is associated with the fact that the combination of external signals is most efficient in selecting the attractor 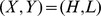 in the face of fluctuations when the rising times 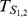 are larger (for a constant maximum asymmetry 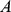 respecting Eq. (1), where 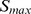 stands for the maximum amplitude allowed for each external signal). This is a consequence of larger 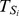
’s corresponding to smaller sweeping speeds through the critical region.

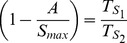
(1)As in canonical models of nonequilibrium statistical physics [6] or dynamic bifurcations [7], the probability that during the sweeping process the system forced by noise jumps across the potential barrier located at the basin of attraction boundary separating the desired end state 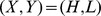 from 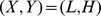, is reduced when the system goes slowly through its critical region.

**Figure 2:**
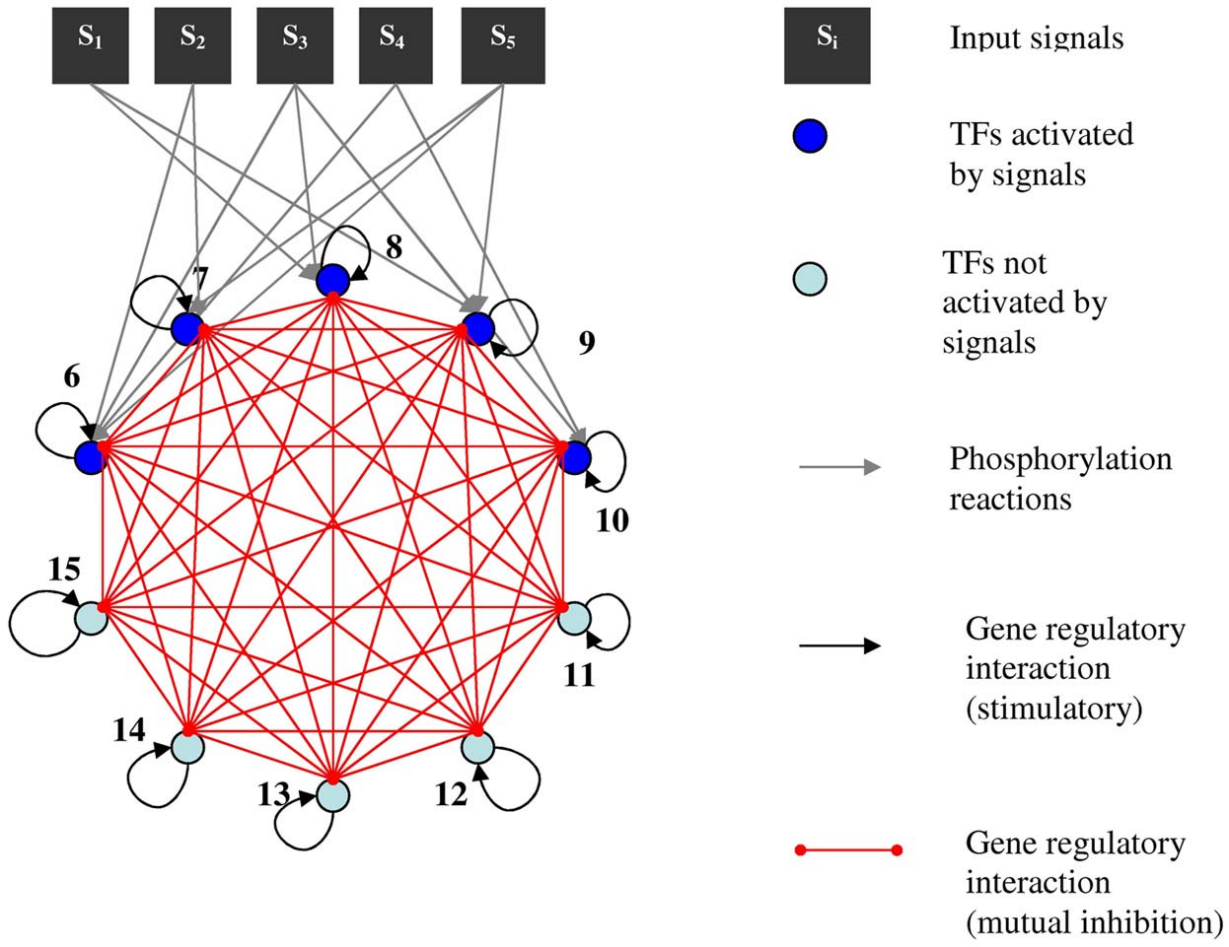
Representation of the high-dimensional genetic decision switch with external stimulation. Nodes 6 to 15 represent proteins, transcription factors. Signals 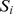 represent protein kinases. Only nodes 6 to 10 need to be activated (phosphorylated) to act on any promoter region of the rest of the transcription factors in the network. Each transcription factor reinforces its own expression (black arrows) and represses (red links) all other nodes. Phosphorylation reactions are represented by grey arrows. See also figure legend on right hand side.

In the present work, we extend the findings reviewed above, and fully explored in [5], to a high-dimensional genetic switch (see Fig. 2) in the presence of fluctuations (see also Methods). High-dimensional switches have been used before to model generalized, switch-like competitive basic Helix-Loop-Helix heterodimerization networks in the context of differentiation [8]–[10]. A set of rules for the clustering capacity of this type of network was devised as a function of competition between synthesis, degradation and complex formation rates of different elements. In our work we will focus on a specific type of network parameters that induce multistability but in a different class of models (see Methods) from those previously explored in [8]–[10].

The high-order transcriptional circuit chosen will be stimulated by a set of external signals 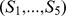 (see Fig. 2) driving gene expression, a common assumption in gene regulatory network models [2], [4], [5]. For simplicity, each external signal combination 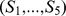 will only differ on their rising times 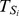 (see for illustration purposes the example presented in Fig. 1B for the low order circuit). As with the bistable switch previously studied [5], the differences in rising times impose time-dependent asymmetries which are processed by the network. Unlike the low order decision genetic switch, here we additionally consider an extra layer of nodes 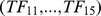 that should respond to the activity of the “genomic gateway” set of nodes 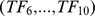 (see Fig. 2). We chose to work with five inputs because it stands as the number of nodes most often associated in the literature with competing attractor selection by signals [11]. Usually, the external signals studied are: Akt, whose activity has been correlated with apoptosis; Erk, which is linked with proliferation; Rac, which regulates the cytoskeletal activity; Sapk and p38, which are cellular stress related nodes [11]. For simplicity purposes and in order to generalize the structure of the genetic switch studied before [5], we limited the number of nodes to five in both layers of transcription factors represented in Fig. 2. An important feature of our model is the fact that only half of the transcription factors (from 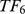 to 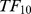, see Fig. 2) need to go through an activation reaction before being able to act on a downstream promoter region. This models generically the action of signaling molecules on Immediate Early Gene products (IEGs) such as c-jun, c-fos and c-myc [12]. The rest of the transcription factors (from 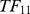 to 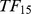, see Fig. 2) operate even if no signal is present. They stand for Delayed Early Gene products (DEGs), the second wave of transcription initiated by the signal [12]. Although this scenario is a condensed approach to modeling the interface between the signaling module and the transcriptional machinery, it serves our objective: observe and generalize the effects of parameter sweeping speed and transient external asymmetries on high-dimensional attractor selection in phase space, here equated with the space of concentrations of each of the transcription factors.

The combinations of external signals are expected to be associated with particular transcriptional programs [1], [12], [13]. The progression from an initial state or phenotype to the outcome of cell fate decision is performed by a sequence of steps or path in phase space [1], [2], [14]. This path is determined to an extent by 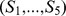 (see Fig. 2), in the case of our model. Due to the fact that gene expression is affected by fluctuations [15], the path forced by the external inputs may suffer substantial alterations which may affect cellular decision making. Therefore, not only external signal amplitudes and duration [4], [16], [17] but also their shapes determined by rising and decay times may become relevant.

## Results and Discussion

### High-dimensional Regulatory Network Exhibits Multistability

An extensive study of all sets of parameters (see Methods and Table 1) and 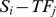 connectivity matrices (with 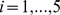 and 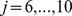) was performed for the high-dimensional genetic switch. We selected the network that exhibited the highest number of attractors in phase space in order to generate, potentially, maximum discrimination between combinations of inputs. The resultant connectivity between the set of signaling inputs 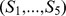 and the set of transcription factors activated by phosphorylation (see Fig. 2) was the following (see Eq. (2)):
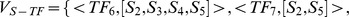


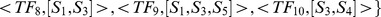
(2)


Each link between 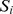
’s and 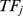
’s (see Eq. (2)), with 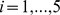 and 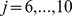, is stimulatory. As in the study performed on the low order genetic switch with external stimulation [5] (see also Fig. 1), we will focus on the bias produced by the set of external signals 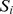 stimulating the high-dimensional genetic switch. Therefore, any parameters representing activation or transcription and translation of proteins will be assumed to be equal for each transcription factor node in Fig. 2 (see also Methods and Table 1).

**Table 1:** Parameters in the high-dimensional decision genetic switch with external stimulation model.

Parameter	Interpretation	Value
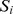	External signal 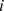	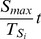 , 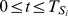 and 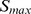 , 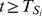
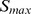	Maximum amplitude of any 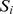	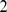
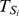	Rising times of 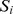	–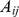	Maximum asymmetry between 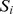 and 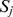	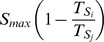
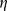	Basal transcription rate multiplied by translation rate divided by 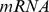 and protein degradation rates	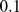
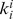	Ratio between binding and unbinding affinities of dimers to promoter regions for self-activation, respectively	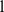
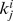	Ratio between binding and unbinding affinities of dimers to promoter regions for cross-inhibition, respectively	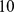
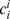	Ratio between rate of expression of the respective gene when homodimers are bound and basal transcription	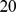
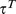	Combined dimensionless time scale for transcription and translation of proteins	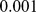 and 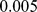
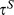	Dimensionless time scale for phosphorylation processes	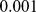
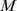	Allowed order of dimers, homo and hetero, in the high-dimensional genetic switch model	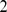
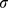	Intensity of Gaussian noise 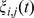 with zero mean and 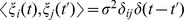	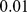 , 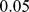 and 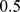

Parameters used in Eqs. (6) to (10) and their respective interpretation and values. See also [39].

**Figure 3:**
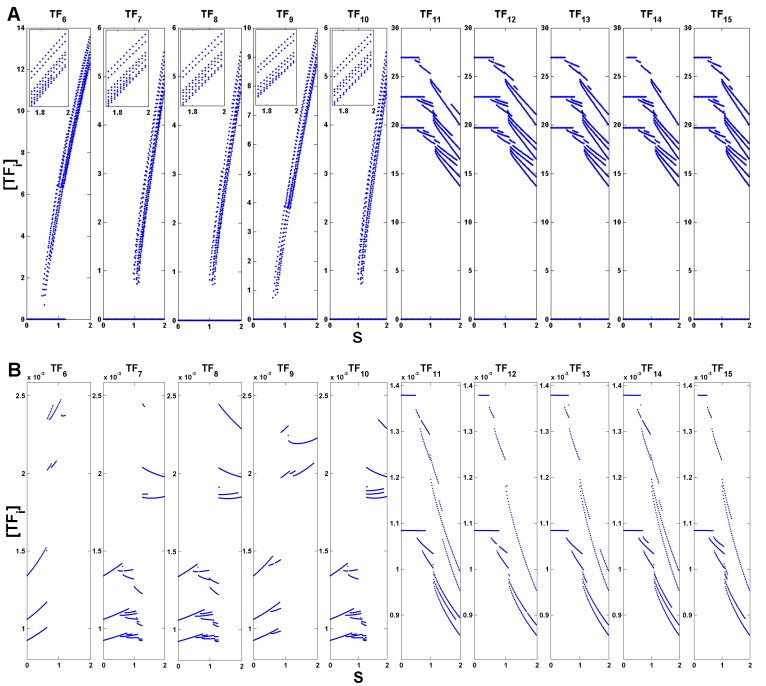
Bifurcation diagram for each of the transcription factors for 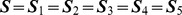. (A) Complete bifurcation diagram. Inset: detail of branches near 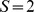. (B) Amplification of states represented in (A) close to zero. Parameters: 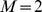, 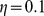, 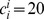, 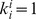 (self-activation) and 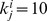 (cross-repression), 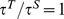, for 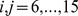 (see Methods). 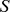 is the horizontal axis for all the figures, from 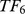 to 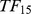. 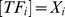, i.e. the concentration of each transcription factor is represented here by 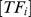 and associated with 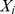 in Eqs. (7) and (8) with 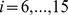 (see Methods). In the construction of the bifurcation diagrams 100 initial conditions were randomly selected for each 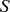 and the long term trajectories recorded and plotted.

The existence of multistability can be verified, for example, in bifurcation diagrams generated by assuming 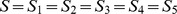 (see Fig. 3A). For each value of critical parameter 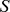 the attractors emerging from initiating the system at 100 random initial conditions were recorded and plotted (see also Methods for the equations behind the computations performed). One can clearly verify the existence of multiple attractors for all network nodes. For the set of nodes activated by the external signals 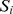, i.e. 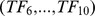 (see Fig. 2), only when the signal amplitude crosses a certain threshold, 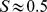 for 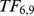 and 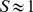 for 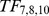, do multiple attractors above zero become clear. Actually, even before the amplitude reaches this point there’s a very fine set of states very close to zero (see Fig. 3B). For the remaining set of transcription factor nodes that do not directly interact with any 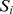, i.e. 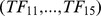 (see Fig. 2), the existence of multiple high concentration stable states is clear even for low values of parameter 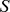. Additionally, there is also a very fine set of attractors very close to zero for nodes 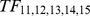 (see Fig. 3B). As the control parameter 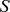 is raised the nodes from 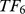 to 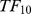 tend to show higher and higher stable state concentrations. Nevertheless, a set of low concentration steady states is still observed for all values of 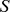 and for all nodes with the exception of 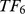. Regarding the nodes from 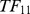 to 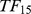, higher levels of 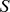 reduce the stable state concentration levels (Fig. 3A). The finer structure of stable states close to zero is also maintained for this set of transcription factors (Fig. 3B).

The bifurcation diagrams in Fig. 3 show that for the chosen set of parameters the system seems to go through a subcritical type of bifurcation, due to the disconnection between emerging branches. Indeed, this class of models and set of parameters has shown to induce in 2 dimensional genetic switches a transition between a region of 1 stable state with low concentration values, and another with three stable states with high concentration values [4]. Although the model in [4] was slightly different (only homodimers were allowed), if a similar process is present in our circuit then the disconnection is indeed caused by a subcritical type of bifurcation. On the other hand, the type of bifurcation present may be supercritical and further sampling of the state space is necessary to dismiss other options. Although the mechanism of SdCDM has been explored in supercritical systems and relies on both the intrinsic dynamics of the system and the dynamics of the external driving signal near the bifurcation point [5], [6], subcritical systems may also reveal speed-dependent effects when control parameters are made time-dependent [18].

For the time-dependent external signals studied ahead, the asymmetries 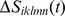 (with 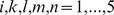) between each of the inputs influence the available attractors in the system at each time step, as was the case of the small genetic switch studied in [5] and summarized in Fig. 1. Further ahead we will focus on three specific input combinations. Their bifurcation diagrams show relatively small differences (compare Figs. S1, S2 and S3). Yet, as will be seen in following sections, this is sufficient to induce differences in long-term distributions over stable states when fluctuations are considered.

### Clustering of Input Signal Combinations

In order to understand if differences in time-dependent input signal profiles force the system to converge to different attractors, we tested the response of the high-dimensional decision switch to a batch of 100 combinations of inputs, 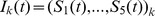, generated by randomly selecting 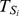
’s (see Fig. 1B for illustration purposes) for each input 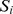. The maximum amplitude 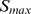 allowed for each signal 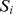 was 2. This value arose from the initial investigations that led to the choice of a set parameter values (see Table 1) and 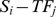 connectivity matrices, with 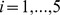 and 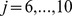 (see Eq. (2)), that generated the highest number of attractors. For each combination 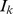 the system was randomly initiated at 100 initial conditions, with 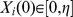, for 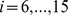 (see Methods and Table 1). Subsequently, the asymptotic stable states were recorded for each of the combinations 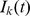 and each of the initial conditions long after the largest 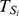 had been reached. For all input combinations the set of initial conditions was exactly the same.

In order to quantify the differences in the number of trajectories converging to each stable state forced by each combination 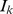, the average Euclidean distance (AED, see Eq. (3)) between the set of concentrations 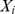, in the limit of large times, was compared for all possible pairs 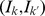 and averaged over the number of initial conditions tested (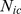 in Eq. (3)). Further investigations will be performed in subsequent studies by applying other distance metrics in high-dimensional phase space, e.g. the ISOMAP [2], [19] or extensions thereof [20]. Here we must stress that the bifurcation diagrams shown in Figs. 3, S1, S2 and S3 represent only the available stable states at each amplitude of the external signals. When time-dependent signals are considered the configuration of the phase space changes with time. Despite the fact that the available stable states for each amplitude, at each time instant, are the same as those determined in the respective bifurcation diagrams, the dynamics arising from changing the phase space in time will not be the same as that arising from holding the signal amplitudes at a certain level and letting the system converge to its asymptotic state. Further analysis is necessary to quantify exactly the differences in the dynamics stemming from both situations. Here, we will focus only on the end state of the sweeping process. We will assert if possible differences in the dynamics arising from a phase space changing with time result in significant changes in the selectivity of attractors.
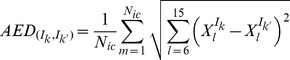
(3)


**Figure 4:**
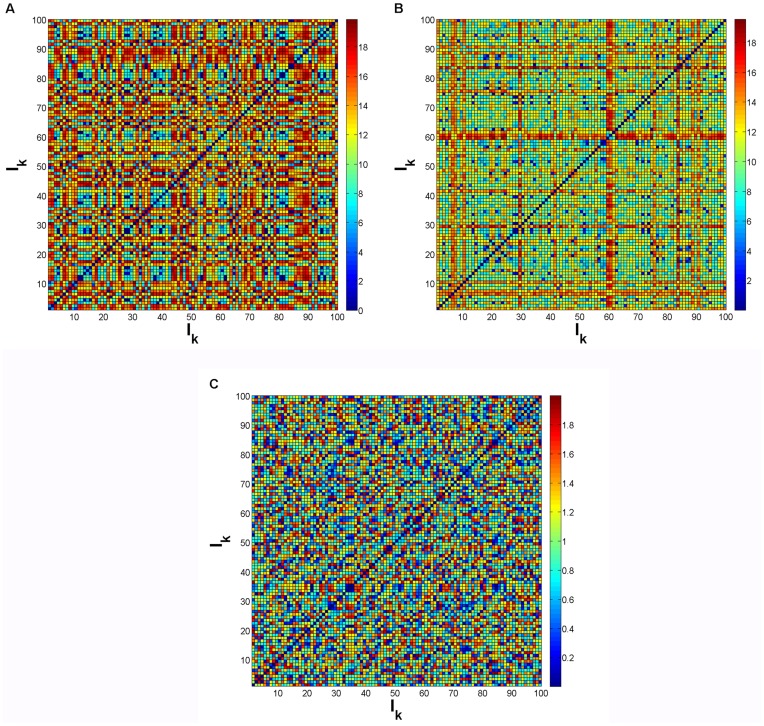
Pair-wise average distance between asymptotically stable states induced by input combinations. (A) Results for time scale ratio 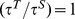 calculated through Eq. (3) and (B) 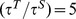. (C) Distance between pairs of vectors 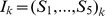, calculated through the distance metric 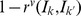, with 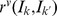 being the Pearson coefficient of correlation between the actual vectors 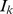 and 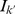. Parameters: 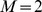, 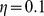, 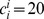, 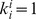 (self-activation) and 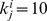 (cross-repression)(see Methods), for 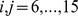.

In Fig. 4A and B, the results obtained from the application of Eq. (3) can be visualized for two time scale ratios 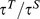 (see Methods and Table 1). Because the matrices presented are symmetric we need only to observe values below the diagonal. In both matrices one can verify that certain pairs 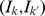 force the system to converge to different attractors even if the initial conditions and the initial and final amplitudes for each 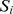 are the same (red pixels, higher 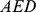 distance). Others, for the same initial conditions, select exactly the same attractors, on average (blue pixels, lower 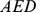). This indicates that certain combinations 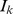 of signals 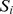 are clustered together due to the incapacity of the network to memorize the transient asymmetries 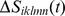 (with 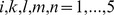) intrinsic to each of them. In order to verify if the pairs 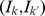 inducing the same attractors were doing it because their differences were very reduced, we calculated the distances between the input vectors 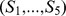 corresponding to each pair of input combinations (see Fig. 4 C), by applying a correlation based metric. By visual inspection (see for example Fig. 4A and C) we can conclude that no clear correlation exists between the distance between input vectors 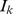 and the average euclidean distances (AED, see Eq. (3)). Indeed, the correlation between the vectors obtained by concatenation of the lines of each of the matrices represented in Fig. 4A and C, and Fig. 4B and C, is 0.1283 and 0.1588, respectively.

Observing Fig. 4B we see that overall the AED distance (Eq. (3)) for each pair of input combinations is decreased if the time scale ratio (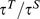) (see Methods) of transcription over phosphorylation processes is raised. This effect had been seen already in the low-order decision genetic switch [5], although in the presence of fluctuations. In real biological systems the time scale differences between phosphorylation and transcription reactions can be substantial [21]. If genetic circuits are not sensitive to slight differences between driving external signals when time scale separation is significant, then integration of signals is only successful when very pronounced external asymmetries occur. Ultimately, only considerable differences in amplitude held for an interval compared to the characteristic relaxation time scale of the system will be discriminated efficiently.

### Path-dependent Effects on Attractor Selectivity in the Presence of Multiplicative Noise

In order to prove the existence of path-dependent effects in attractor selectivity in the presence of fluctuations, first we analyzed the inter-trajectory distance for every pair (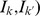 generating the same end attractors when 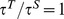 (see Fig. 4A, dark blue pixels) and noise intensity is zero. For this calculation we used the correlation based distance metric 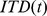 represented in Eq. (4), where 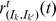 stands for correlation between trajectories induced by vectors 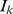 and 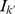. Throughout our work selectivity represents the fraction of trajectories in a stochastic simulation that converge to a specific attractor.
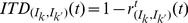
(4)


**Figure 5:**
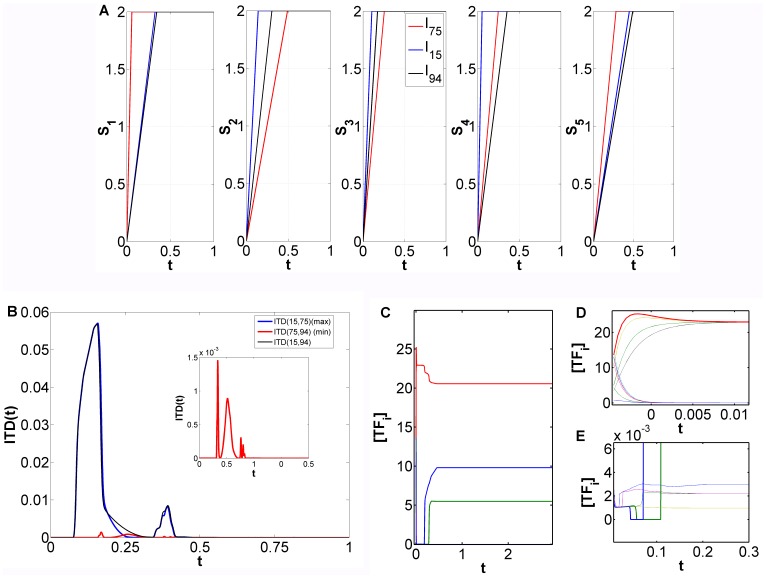
Inter-trajectory distance, profile of specific input combinations and typical switching dynamics. (A) Time-dependent profile for each input 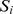 for 3 input combinations: 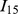, 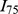, 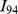. (B) Inter-trajectory distance for pairs 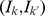 inducing the same attractors (see Fig. 4A). Pairs exhibiting the highest value for 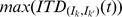 (Eq. (4)) and the lowest value for 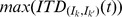. Inset: zoom of 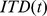 curve for 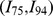. (C) Typical evolution of concentrations for all the nodes 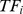, 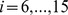. This particular trajectory was generated by applying 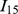 and noise intensity 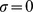 (see Methods). (D) Amplification of (C) for early times t. (E) Amplification of (C) for concentrations 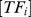 close to zero. 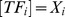, i.e. the concentration of each transcription factor is represented here by 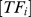 and associated with 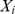 in Eqs. (7) and (8) with 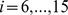 (see Methods). Parameters: 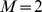, 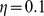, 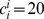, 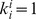 (self-activation) and 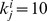 (cross-repression), 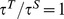 (see Methods), for 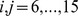.

The pair 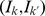 with input combinations inducing the same end attractors that had, at a particular instant, the highest maximum for the inter-trajectory distance 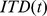 (Eq. (4)) amongst all the pairs was 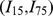 (see Fig. 5B). On the other hand, the pair exhibiting the smallest maximum was 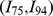 (see Fig. 5B). The time-dependent profiles for 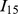, 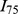 and 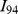 can be visualized in Fig. 5A. A typical trajectory in time can also be observed in Fig. 5C. The trajectory presented corresponds to the evolution of the system by applying 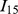. Yet, it represents the typical dynamics observed for any input combination 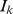, the only difference being the allocation of nodes per stable state. Regarding the switching dynamics, usually the trajectories converge very rapidly to high or low concentration values (Fig. 5D). Subsequently, for nodes migrating to low concentration values there is a further reorganization of states (Fig. 5E). In the vicinity of the instant when all 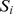
’s have reached their maximum amplitude there’s further reorganization of states with certain nodes reaching intermediate concentration values (see Fig. 5C and E). Although for the example shown in Fig. 5C it is not clear the existence of multiple attractors at high concentration values, these do exist as can be visualized in the bifurcation diagrams of Figs. 3, S1, S2 and S3.

**Figure 6:**
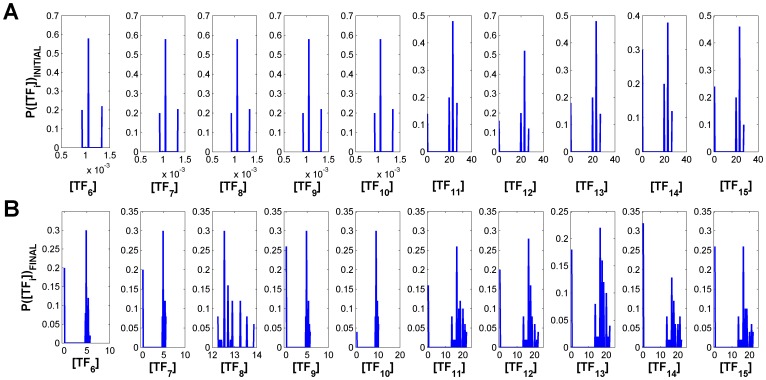
Initial and final attractor frequency in the absence of fluctuations. (A) Attractors available for 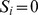, with 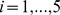, and respective frequency. (B) Attractors available for 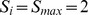, with 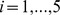. The frequency of the attractors shown here will change when each of the selected input combinations is applied in the presence of fluctuations. This stems from path-dependent effects on attractor selection (discussed in main text). 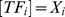, i.e. the concentration of each transcription factor is represented here by 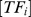 and associated with 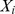 in Eqs. (7) and (8) with 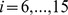 (see Methods). Parameters: 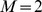, 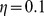, 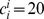, 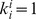 (self-activation) and 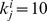 (cross-repression), 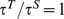, 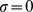 (see Methods), for 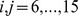.

The probability of each attractor when all 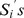 are held at an amplitude of 0 and 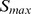 can be seen in Fig. 6. One should remember that each of the selected combinations 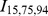 has exactly the same initial and final signal amplitudes. Therefore the phase space looks exactly the same. If any differences arise due to path-dependent effects forced by the time-dependent asymmetries 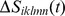, then the frequencies observed for each attractor when the selected input combinations are applied will be different (discussed ahead). Fig. 6 was obtained by collecting the stable-state values for the concentration of each 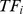 (see Fig. 2) starting at 100 initials conditions, and in the absence of noise.

One can observe that, when every 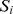 is equal to 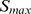, the nodes 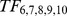 (Fig. 6B) show propensity to converge to attractors with intermediate and high concentrations. Regarding this set of nodes it is possible to verify that there is also some probability of reaching attractors close to zero. These low concentration attractors are very close to each other (see Fig. 3B). One should add that regarding node 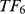 the presence of attractors states close to zero at high external signal amplitudes is inconsistent with what we observed for the bifurcation diagram in Fig. 3. For node 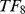 the opposite of what is verified for 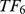 occurs. We must then conclude that this discrepancy arises from initial condition sampling issues. For nodes corresponding to the DEG layer, i.e. 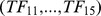 (see Fig. 2), higher selectivity frequencies for most of the nodes are registered for attractors with higher concentrations. However, there is still a high number of trajectories with asymptotic states near zero (Fig. 6B).

The three input combinations 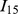, 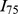 and 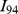 were once again applied to the circuit but in the presence of fluctuations. Overall, the data from 5000 trajectories for each selected input combination was collected, including random starting points in phase space. Several observable changes in the attractors selected were noticed. For the IEG layer of transcription factors, i.e. 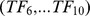 (see Fig. 2), there was a considerable transfer of probability mass to states located near zero (figure not shown). These were not identified as being very probable in the deterministic scenario (see Fig. 6B). The addition of noise forces the system to jump across potential barriers, located at the basin of attraction boundaries, to stronger attractors which, in this case, are closer to zero. As was seen in the one dimensional canonical model [22], according to Kramer’s classical theory [23] the transition time for a system in one dimension to jump across the potential barrier decreases with noise intensity. There are several aspects of the attractor selection process that might be occurring here. First, let us recall the probability distribution shown in Fig. 6. These results are dependent only on differences in attractor basins and number of initial conditions tested. The basin of attraction in dynamical system theory is taken as the percentage of points converging to a specific attractor [24]. Sampling 100 initial points randomly may not have probed completely the phase space. Higher sampling could have revealed finer aspects of attractor basins. A second aspect of the selection process arises as a function of the fact that different externals signals are exerting different changes on the attractor landscape. If the probability mass transfer to attractors located near zero was only a consequence of the combination of input signals, then the differences observed in the presence of noise should have been more pronounced. The only clear differences recorded had very low probabilities (figure not shown). We can conclude from these observations that, although the asymmetries induced by each combination 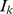 play a significant part in the high frequencies found for low concentration values for the set of nodes 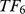 to 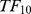, this occurrence is also intrinsically related to the concept of attractor strength. This concept is defined as the minimum size of a perturbation (in our case noise) that results in a very low probability of return [24]. Regarding the frequency of the attractors found for the DEG layer of transcription factor nodes, i.e. 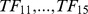, the distribution does not differ considerably in terms of location from that generated in the deterministic scenario. The differences between applying each pair of combinations, 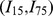 or 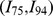, occur mostly in the same set of attractors at high concentration values. Actually, applying one or another input combination shifts the probability maximum to an attractor in the vicinity. We conclude that regarding the DEG layer the differences arising from the application of each of the selected input combinations induces smaller changes in the final distribution of trajectories across attractors.

**Figure 7:**
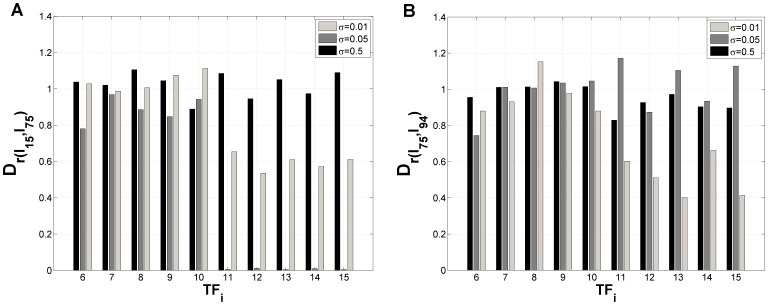
Distance between final distributions generated by different pairs of input combinations 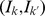 in the presence of fluctuations. (A) Pair 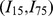. (B) Pair 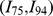. 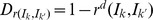 is a correlation based metric, where 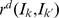 stands for the correlation between the distributions across attractors, induced by 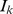 and 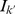, in the limit of large times. Parameters: 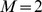, 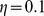, 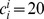, 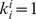 (self-activation) and 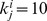 (cross-repression), 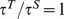 (see Methods), for 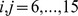. 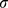 stands for noise intensity (see Methods).

We further evaluated the distance between distributions for several noise intensities (see Fig. 7) to understand if, as in the small integrated signaling-gene regulatory decision switch [5], noise increases symmetry between the distribution across attractors or if its effect is not as strong as previously observed and it only causes new attractors to be populated in conjunction with the changes exerted by each 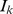. The distance metric 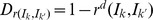 used for the following investigations is a correlation based metric, where 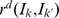 stands for the correlation between the distributions across attractors, induced by 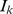 and 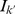, in the limit of large times. For the pair 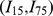, the most noticeable fact when we raised noise intensity from 0.01 to 0.05, is the relative proximity of the distributions for the DEG node layer (Fig. 7A). The 5 fold increment seems to force the system to jump to the strongest attractors. Effectively, comparing by visual inspection the distribution obtained with noise intensity 0.01 and 0.05 (figures not shown), we verified that for noise 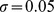 essentially the maximum frequencies for 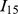 and 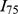 occurred at the same attractors. For the IEG layer of nodes the same observation stands although it was not as evident (Fig. 7A). Raising further the noise intensity increased the distance between final distributions, which was to be expected due to the increased capacity to cross potential barriers and, as a result, populate different attractors. For the pair of input combinations 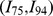 that, as was determined before (Fig. 5), had a very small difference between the trajectories in phase space, the tendency observed for the distance calculated between distributions when noise intensity is increased from 0.01 to 0.5 was similar to that of the pair 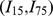. Also, for these noise intensities 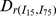 is higher than 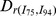, which is consistent with the fact that 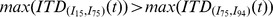 (Fig. 5B). Nevertheless, for noise amplitude equal to 0.05 the tendency observed for 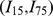 was not maintained. At this noise intensity, instead of an optimal attractor selection that approximates the distributions, the opposite effect is present. The numerical results reported above indicate that there is an optimal intensity of noise that increases the convergence of trajectories to the same attractors, when the differences between trajectories induced by each 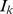 is larger. When the differences in phase space trajectory are small the noise optimality effect observed before reverses its role and increases inter-distribution distance.

### The Importance of Sweeping Speed for High-dimensional Attractor Selection in the Presence of Fluctuations

To test SdCDM [5] in the high-dimensional switch we extended the simulation experiments for noise intensity 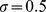. We chose to perform the extra simulations with the maximum noise intensity to understand if the sweeping speed could override the strong effects of noise. The original selected combinations, 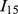, 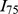, 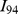, were changed in a way that the maximum asymmetry between each of the inputs 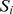 was maintained but the sweeping speed was decreased. The following steps were taken:

1. For input 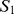 of the original combination calculate the maximum asymmetry reached between 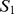 (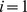) and 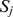 recurring to Eq. (5);

2. Increase 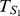 by n numerical integration time-steps and calculate the necessary 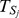 (Eq. (5)) for each of the inputs that maintains the maximum asymmetries 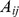 between each of the signals 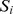 and 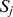.
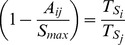
(5)


**Figure 8:**
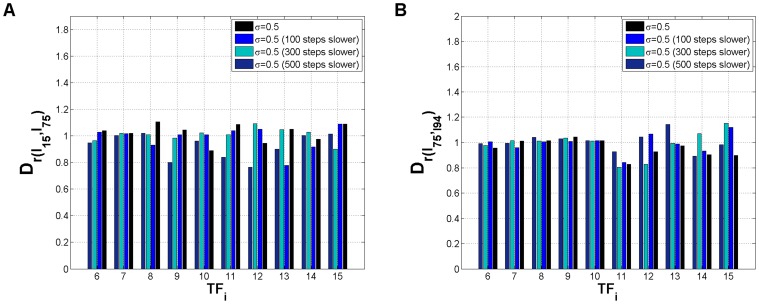
Inter-distribution distance dependence on sweeping speed. (A) Inter-distribution distance between the attractors induced by combination 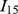 and 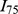. (B) Inter-distribution distance between the attractors induced by combination 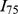 and 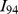. 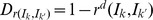 is a correlation based metric, where 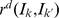 stands for the correlation between the distributions across attractors, induced by 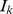 and 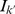, in the limit of large times. Parameters: M = 2, 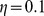, 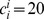, 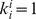 (self-activation) and 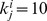 (cross-repression), 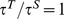 (see Methods), for i, j = 6,…,15. 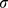 stands for noise intensity (see Methods). On each figure each color corresponds to different sweeping speeds obtained by increasing 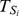 by 100, 300, or 500 numerical integration time-steps.

This strategy secures that the signals induce similar changes in the transcriptional landscape as the original combinations, but at a smaller speed. The distance between the final distributions was calculated again by applying a correlation based distance metric to three extra cases: same input combinations but 100, 300 and 500 numerical integration time-steps slower. The results are shown in Fig. 8. In light of the results obtained for the small genetic decision switch [5] (see also Fig. 1) we expected that the differences between final distributions across attractors induced by each pair 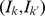 would be increased if the speed with which the signals 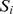 are changed is reduced. Figure 8 shows that, overall, the path-dependent effects registered before for the pair of input combinations 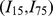 are less clear if we perform the sweeping process at lower rates. Comparing with the original results (black bars, Fig. 8A), we can verify that by decreasing the sweeping speed through the bifurcation region (by imposing for example 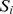
’s 500 time-steps slower) seems to have, for most of the transcription factors, an effect which brings the distributions induced by 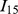 and 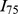 closer together. For the other sweeping speed experiments (Fig. 8 A, 100 and 300 steps slower) there seems to be a tendency for the pair 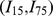 to induce closer and closer final distributions as we decrease the sweeping speed. Yet, this occurs in a non-monotonous fashion. This observation contrasts with the findings of speed-dependent decision making in the bistable decision genetic switch (see Fig. 1) where slower sweeping rates increased the sensitivity to external asymmetries. The differences in the final distributions arising from the respective paths in phase space should have been more pronounced. On the other hand, we do observe reasonably clear speed-dependent effects for the high-dimensional switch, although with a different outcome from that of [5]. Further simulation studies, for 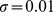 and 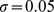, are necessary to clarify the synergistic effects of sweeping speed and noise intensity in high-dimensional phase space with irregular attractor landscapes. Regarding the other input combination pair, 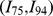 (see Fig. 8B), a considerable reduction in sweeping speed (500 time-steps slower) induces exactly the opposite effect observed for 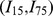. This tendency to observe opposite effects in the input combination pairs used throughout this work is quite intriguing and should be investigated with the complete set of pairs 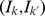 with same end attractors (see Fig. 4). Overall, we observe that slower sweeping speeds induce a higher sensitivity of the high-dimensional circuit to external signals when the differences between the respective paths in high-dimensional phase space, induced by each pair 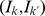, are smaller.

The generalization of the parameter sweeping mechanism to high-dimensional space demonstrated that it is dependent on phase space structure and the efficiency of noise to induce transitions across potential barriers. Moreover, the capacity of high-dimensional genetic circuits to integrate a combination of complex signals is closely linked to the initial condition chosen. It was also clearly shown that input combinations that generate the same attractors in a deterministic system have significant differences in the final distributions when noise is taken into account. Hence, path-dependent effects exerted by different complex signals and noise are relevant for attractor selectivity and cell fate decision in high-dimensional systems. We have also shown that the speed of signaling in genetic switches changes significantly the result of cellular decision, an effect that we had termed speed-dependent cellular decision making (SdCDM) [5], and that it is also relevant in high order circuits. In contrast to other aspects of nonequilibrium physics [25]–[27], dynamic bifurcations have only recently been systematically studied in systems biology [5], [28]–[30], despite involving fundamental aspects of cell fate decision. It is of special interest in this context because all genetic switches are asymmetric and stochastic and, hence, can be expected to demonstrate both path and speed-dependent effects in the process of phenotype selection. Additionally, certain cell differentiation processes have been demonstrated to be driven by slow build-up of decision-driving signals [31] and experiments have revealed that temporal competition can determine cell fate choice in multipotent differentiation [32], thus indicating a predominant role of time-dependent effects.

Regarding the response of the DEG layer of nodes, 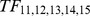, to IEG products, 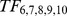, or even external signals 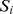 (see Fig. 2), recent studies have shown that the function of regulators in the immediate early response “may be used to put the cell into a transient receptive state…by moving the system out of its attractor basin” [33]. In our model this stage arises from the dynamics of the nodes activated by signals. Although further studies are necessary to understand the mutual information between immediate early dynamics and the delayed responses, we should add that the immediate early response not only puts the system in a receptive transient state, but also induces time-dependent changes on the transcriptional landscape in order to generate the correct, or most probable, decision outcome.

Further studies are necessary to understand speed-dependent attractor selection in systems which consider additional inter-cellular connections and thus show coexistence of different dynamical regimes [34], [35]. This endeavor will constitute an interesting extension and contribute to the clarification of real selectivity mechanisms present in cells that execute competing differentiation, proliferation and apoptosis programs. Additionally, SdCDM in spatiotemporal pattern formation could also play a crucial part in the self-organized, stochastic, gradual patterning behavior observed for instance in paradigmatic inter-cellular phenomena arising in development [36]. One can hypothesize that evolution has selected for embryonic development with the optimal cellular differentiation speed. The conditions leading to deviations from this optimal route, the onset of pathologies and their possible treatment, constitute still an important open question. Speed-dependent decision making effects in biological systems contributes to the area of critical transitions in open systems [37], so crucial for the understanding of selectivity mechanisms in a wide range of subjects [38].

## Methods

The dynamics of the protein concentrations involved in our circuit (see Fig. 2) is described by a phenomenological model following [39] and assumed to be dimensionless. The variables 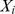 or 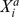 (see Eqs. (6) to (10)) represent the concentration of transcription factors, i.e. 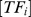, in their inactive and active forms, respectively. For each 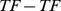 connection, associated with a protein-gene interaction or regulatory process (see Fig. 2), we resorted to a generic representation shown in Eq. (7) and (8). All regulatory interactions to any gene are replaced with an average or effective interaction, taking into account the repression, activation and multimerization mechanisms inherent to epigenetic regulation. This formalism stands as a generalization of [5] but takes into account all possible reactions between input nodes and allows for both hetero and homodimers (see Eqs. (9) and (10)).
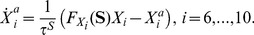
(6)

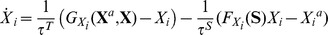


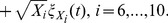
(7)

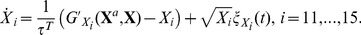
(8)


In this model, Eq. (6) represents activation of transcription factors by phosphorylation-dephosphorylation [12], where the latter is assumed to occur with a constant rate (corresponding to a constant phosphatase concentration, a common assumption in pathway modeling [40]). Phosphorylation is considered to depend on the external signals: 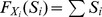, where the sum is done according to the network connectivity set in Eq. (2). In Eqs. (7) the transcriptional input of 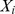 contains the stimulatory action of its phosphorylated form 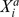 and the inhibitory effect of 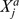, with 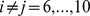, and 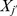, with 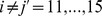 (see Eq. (9)):
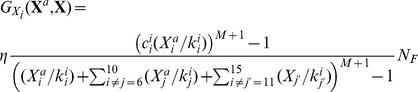
(9)In Eq. (8) the function 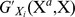 has a similar formula to Eq. (9), although one has to adapt the term to the fact that the transcription factors from 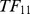 to 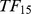 do not need to be phosphorylated to operate on their promoter regions or on other nodes’ (see Eq. (10)):
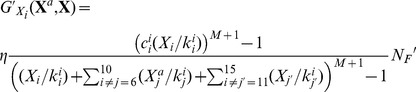
(10)The parameters 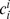 represent the ratio between the maximally activated expression rate and basal transcription, while 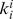 and 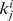 denote activation and repression thresholds. The parameters 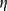 are a measure of the promoter strength multiplied by translational efficiency [39] (see also Table 1). Equation (9) is a simplification of the original input contemplating the action of multimers up to order M [39] where 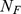 stands for



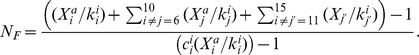
For Eq. (10) similar observations stand and 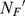 has a formula consistent with Eqs. (8) and (10).

We chose to use the class of models described above due to its compact way of dealing with the complex set of reactions inherent to the transcription initiation process. The larger the multimer order, the larger the cooperativity between input species. Depending on the order M of multimers allowed to be formed, several regimes can be generated by combining both negative and positive links between transcription factors: multiple clustering attractors 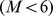, oscillations 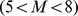 and chaotic regimes 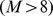 [39]. In the case of the high-dimensional switch chosen for our work, 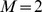, and only a high density of multiple stable states are observed (see Figs. 3, S1, S2 and S3).

Eqs. (6) to (10) were derived by assuming that transcription factor binding and unbinding, on the one hand, and 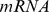 dynamics, on the other, are fast when compared to protein dynamics [4], [21], [39]. Although there is also a substantial difference between the time scales of translation and phosphorylation events [21], the profile of activation of each transcription factor or of signals 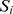 has been demonstrated to be fundamental to understand cell fate decision [16], [17], [41]. Therefore, we maintained the activation Eqs. (6). Moreover, this option allows us to extend in further studies the impact on cell fate decision, here equated with attractor selection, of partial inhibition of phosphorylation processes exerted by specific classes of drugs [42].

Our model assumes that the circuit operates in a constant-volume cell, but takes into account stochastic fluctuations in gene expression [15], through the terms 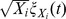 (see Eqs. (7) and (8)) [43]. To that end, 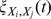 represents a Gaussian noise with zero mean and correlation 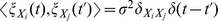, and models the contribution of intrinsic random fluctuations inherent to transcription and translation processes [44] (see Eqs. (7) and (8)). This multiplicative noise term is interpreted in the Ito sense, which is the correct stochastic interpretation for a noise term arising from stochastic protein-gene interaction events [23], [43]. Here we will not consider extrinsic sources of noise such as fluctuations in kinase or phosphatase numbers (see [5]).

### Numerical Methods

All simulation results were performed by numerically integrating the stochastic differential equations using the Heun method [45] with a scaled time-step of 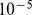. In order to determine each of the quantities represented in the figures shown throughout this work, the set of simulations was performed until an instant far beyond the maximum of each of the rising times for each of the signals 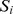 in order to secure that the system had converged.

## Supporting Information

Figure S1
**Bifurcation diagram obtained by setting the parameters 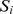 following the combination of amplitudes inherent to 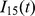.** (A) Complete bifurcation diagram. Inset: detail of branches near 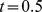. (B) Amplification of lower part of the bifurcation diagram represented in (A). Parameters: 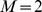, 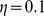, 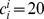, 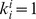 (self-activation) and 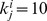 (cross-repression), 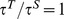 (see Methods) for 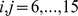. The available attractors at specific times can be visualized. The input combination changes the attractor landscape with respect to the original bifurcation diagram with 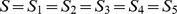 (see Fig. 3) and the other input sequences 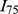 and 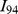. t is the horizontal axis variable for all the figures, from 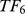 to 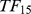. 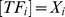, i.e. the concentration of each transcription factor is represented here by 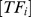 and associated with 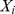 in Eqs. (7) and (8) with 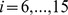 (see Methods). For each time instant t 100 initial conditions were sampled and the respective end attractors recorded and plotted.(TIFF)

Figure S2
**Bifurcation diagram obtained by setting the parameters 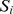 following the combination of amplitudes inherent to 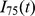.** (A) Complete bifurcation diagram. Inset: detail of branches near 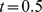. (B) Amplification of lower part of the bifurcation diagram represented in (A). Parameters: 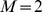, 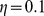, 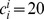, 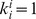 (self-activation) and 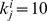 (cross-repression), 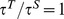 (see Methods) for 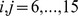. The available attractors at specific times can be visualized. The input combination changes the attractor landscape with respect to the original bifurcation diagram with 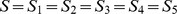 (see Fig. 3) and the other input sequences 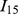 and 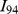. t is the horizontal axis variable for all the figures, from 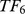 to 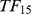. 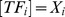, i.e. the concentration of each transcription factor is represented here by 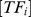 and associated with 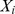 in Eqs. (7) and (8) with 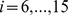 (see Methods). For each time instant t 100 initial conditions were sampled and the respective end attractors recorded and plotted.(TIFF)

Figure S3
**Bifurcation diagram obtained by setting the parameters 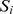 following the combination of amplitudes inherent to 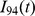.** (A) Complete bifurcation diagram. Inset: detail of branches near 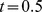. (B) Amplification of lower part of the bifurcation diagram represented in (A). Parameters: 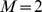, 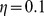, 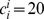, 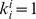 (self-activation) and 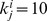 (cross-repression), 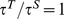 (see Methods) for 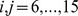. The available attractors at specific times can be visualized. The input combination changes the attractor landscape with respect to the original bifurcation diagram with 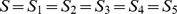 (see Fig. 3) and the other input sequences 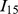 and 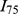. t is the horizontal axis variable for all the figures, from 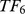 to 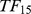. 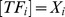, i.e. the concentration of each transcription factor is represented here by 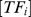 and associated with 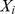 in Eqs. (7) and (8) with 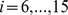 (see Methods). For each time instant t 100 initial conditions were sampled and the respective end attractors recorded and plotted.(TIFF)
