# Supplementary figures and images for: Interplay between Path and Speed in Decision Making by High-Dimensional Stochastic Gene Regulatory Networks
Source: PLoS One. 2012 Jul 16;7(7):e40085. doi: 10.1371/journal.pone.0040085 (PMC3398018; doi:10.1371/journal.pone.0040085)

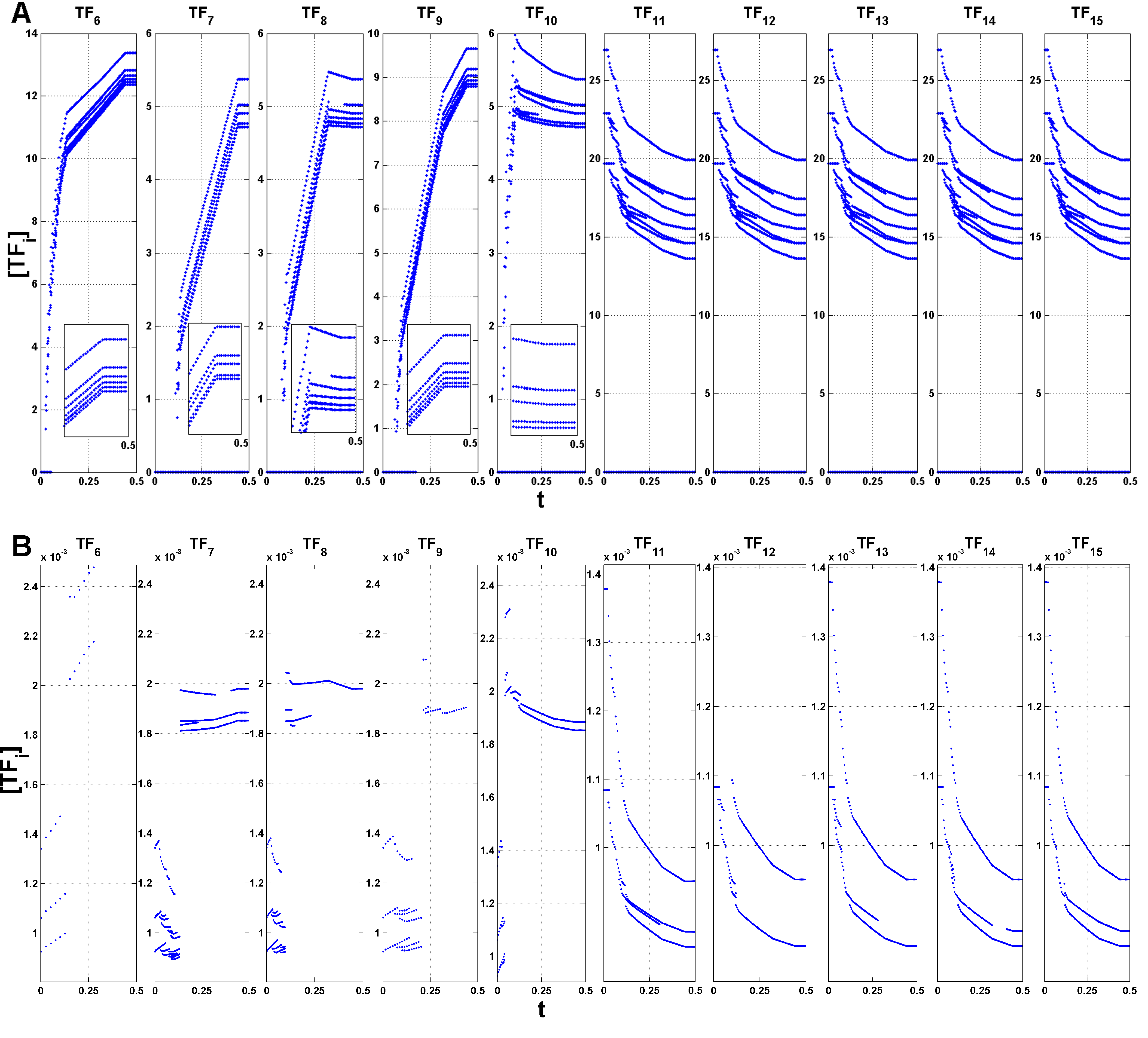

Supplement: Figure S1 — Bifurcation diagram obtained by setting the parameters following the combination of amplitudes inherent to . (A) Complete bifurcation diagram. Inset: detail of branches near . (B) Amplification of lower part of the bifurcation diagram represented in (A). Parameters: , , , (self-activation) and (cross-repression), (see Methods) for . The available attractors at specific times can be visualized. The input combination changes the attractor landscape with respect to the original bifurcation diagram with (see Fig. 3) and the other input sequences and . t is the horizontal axis variable for all the figures, from to . , i.e. the concentration of each transcription factor is represented here by and associated with in Eqs. (7) and (8) with (see Methods). For each time instant t 100 initial conditions were sampled and the respective end attractors recorded and plotted. (TIFF) [file pone.0040085.s001.tiff]

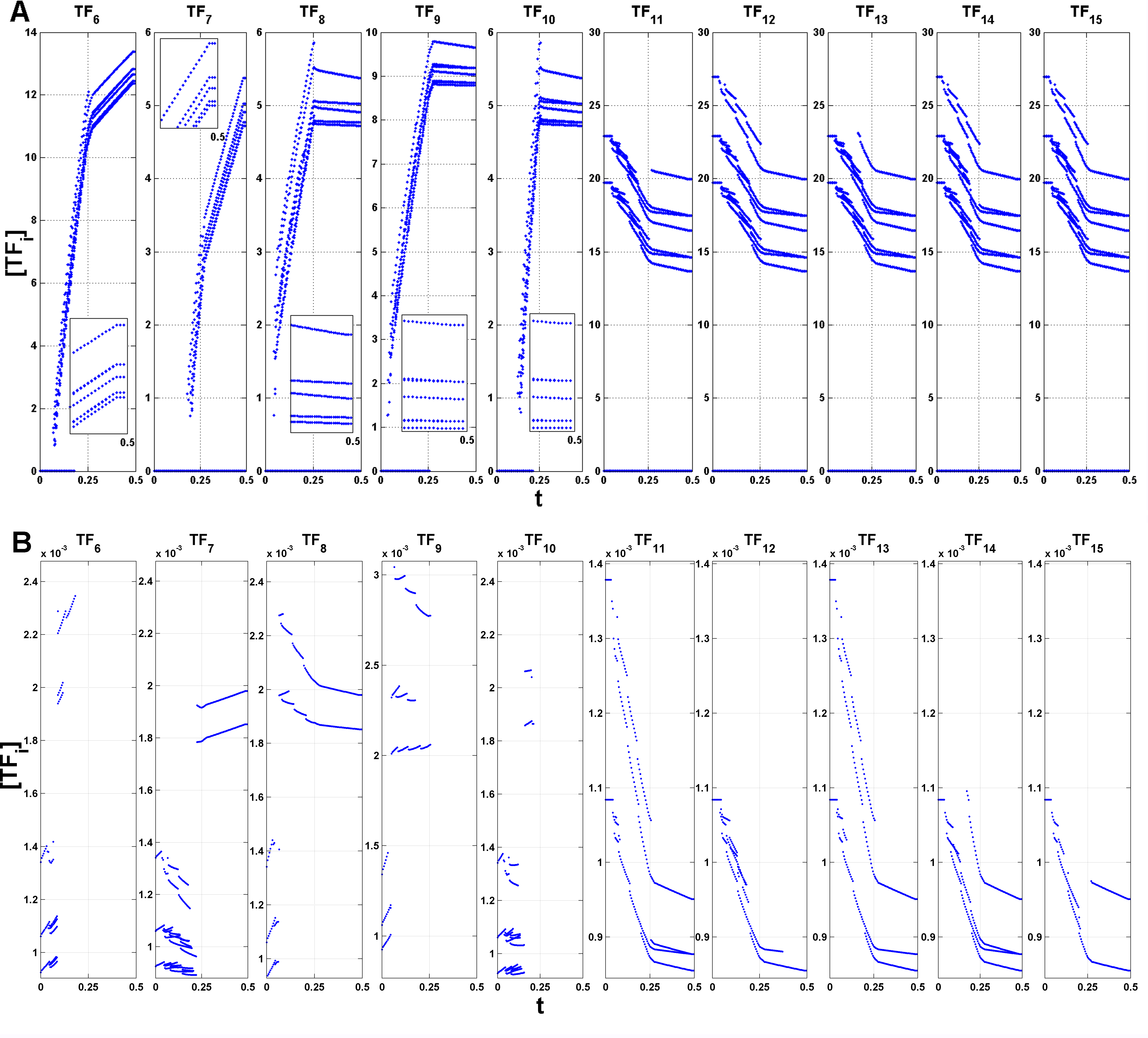

Supplement: Figure S2 — Bifurcation diagram obtained by setting the parameters following the combination of amplitudes inherent to . (A) Complete bifurcation diagram. Inset: detail of branches near . (B) Amplification of lower part of the bifurcation diagram represented in (A). Parameters: , , , (self-activation) and (cross-repression), (see Methods) for . The available attractors at specific times can be visualized. The input combination changes the attractor landscape with respect to the original bifurcation diagram with (see Fig. 3) and the other input sequences and . t is the horizontal axis variable for all the figures, from to . , i.e. the concentration of each transcription factor is represented here by and associated with in Eqs. (7) and (8) with (see Methods). For each time instant t 100 initial conditions were sampled and the respective end attractors recorded and plotted. (TIFF) [file pone.0040085.s002.tiff]

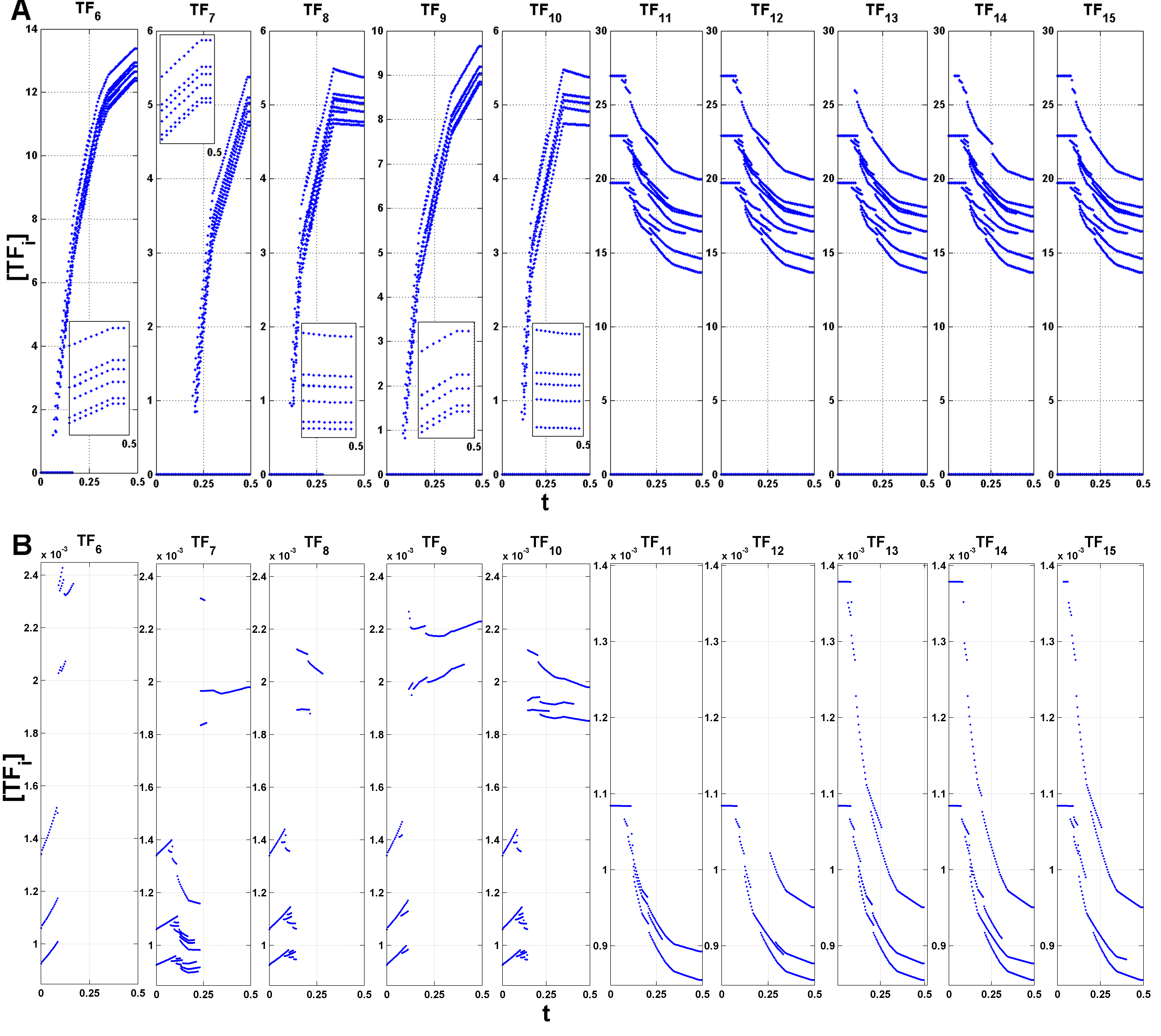

Supplement: Figure S3 — Bifurcation diagram obtained by setting the parameters following the combination of amplitudes inherent to . (A) Complete bifurcation diagram. Inset: detail of branches near . (B) Amplification of lower part of the bifurcation diagram represented in (A). Parameters: , , , (self-activation) and (cross-repression), (see Methods) for . The available attractors at specific times can be visualized. The input combination changes the attractor landscape with respect to the original bifurcation diagram with (see Fig. 3) and the other input sequences and . t is the horizontal axis variable for all the figures, from to . , i.e. the concentration of each transcription factor is represented here by and associated with in Eqs. (7) and (8) with (see Methods). For each time instant t 100 initial conditions were sampled and the respective end attractors recorded and plotted. (TIFF) [file pone.0040085.s003.tiff]
